# Supplementary material for: A synthetic protein as efficient multitarget regulator against complement over-activation
Source: Commun Biol. 2022 Feb 22;5:152. doi: 10.1038/s42003-022-03094-5 (PMC8863895; doi:10.1038/s42003-022-03094-5)
Supplement: Supplementary file 2 — Supplemental material [file 42003_2022_3094_MOESM2_ESM.pdf]

SUPPLEMENTAL MATERIAL

A synthetic protein as efficient multitarget regulator against complement over-activation

Natalia Ruiz-Molina[1], Juliana Parsons[1], Madeleine Müller[1], Sebastian N.W Hoernstein[1], Lennard L. Bohlender[1], Steffen Pumple[1], Peter F. Zipfel[2,3], Karsten Häffner[4], Ralf Reski[1,5], Eva L. Decker[1]*

[1]Plant Biotechnology, Faculty of Biology, University of Freiburg, Freiburg, Germany.
[2]Department of Infection Biology, Leibniz Institute for Natural Product Research and Infection Biology, Jena, Germany.
[3]Institute of Microbiology, Friedrich Schiller University, Jena, Germany.
[4]Faculty of Medicine, Department of Internal Medicine IV, Medical Center - University Freiburg, University of Freiburg, Freiburg, Germany.
[5]Signalling Research Centres BIOSS and CIBSS, University of Freiburg, Freiburg, Germany.

***Correspondence:** Eva L. Decker, eva.decker@biologie.uni-freiburg.de

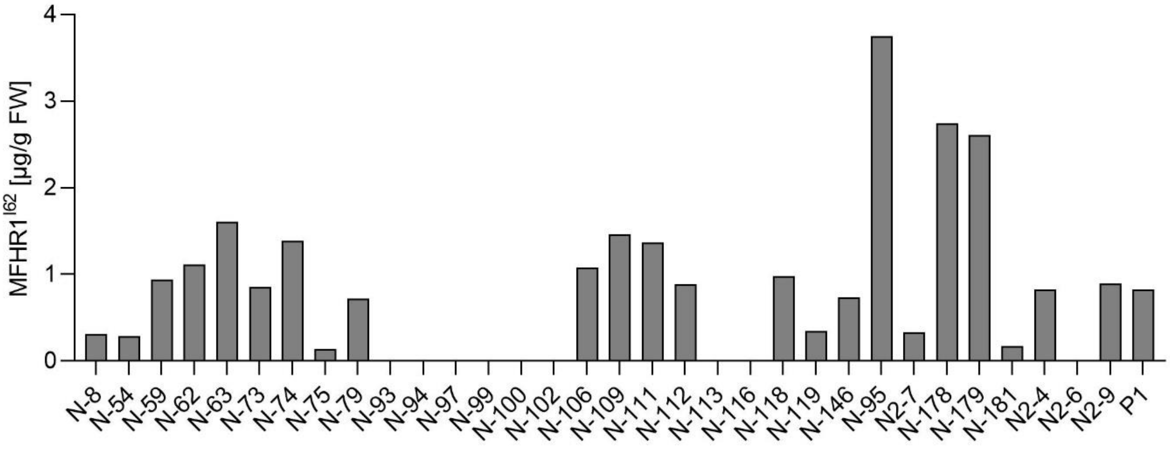

Supplementary Fig. 1: MFHR1^{I62} productivity. Plants surviving the selection were screened for productivity in suspension cultures in agitated flasks via ELISA. P1 is the MFHR1^{V62} moss producer line, which was included as an internal positive control

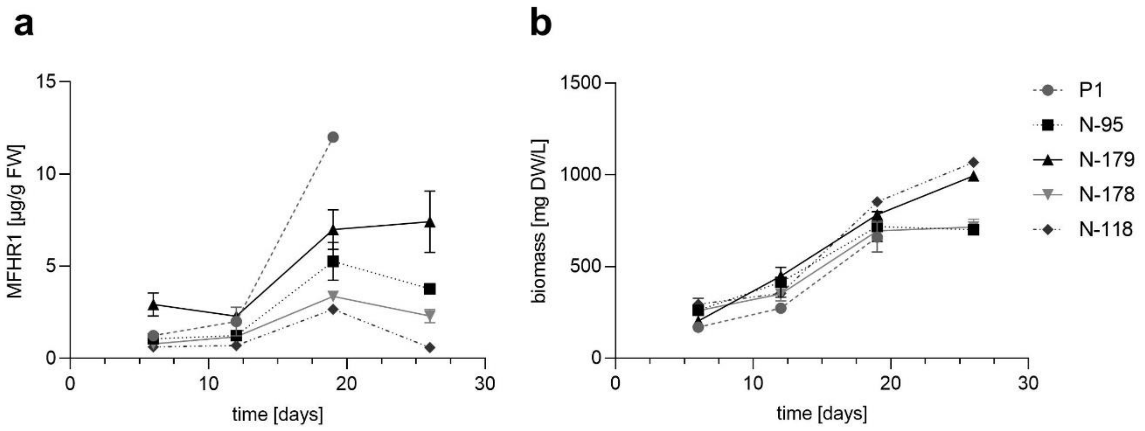

Supplementary Fig. 2: Kinetics of a) MFHR1^{I62} specific productivity and b) biomass accumulation of 4 of the best lines tested in agitated flasks.

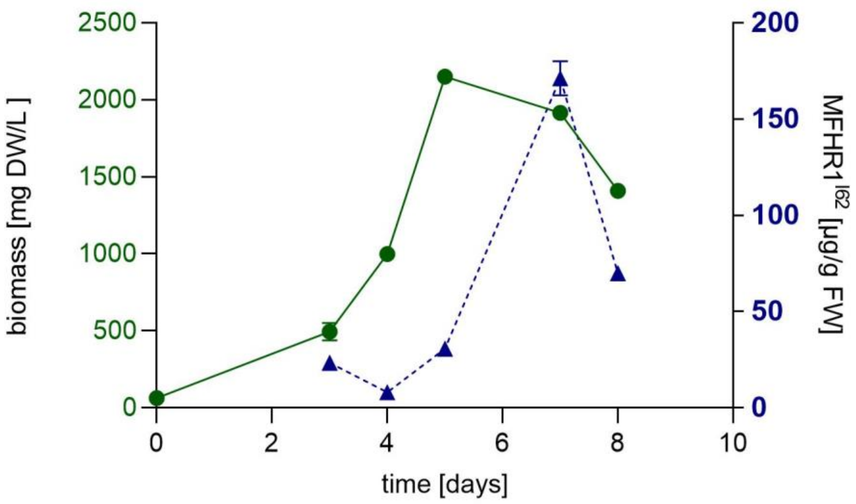

Supplementary Fig. 3: Kinetics of biomass accumulation and MFHR1^{I62} levels of plant N-179 in a 5 L stirred bioreactor.

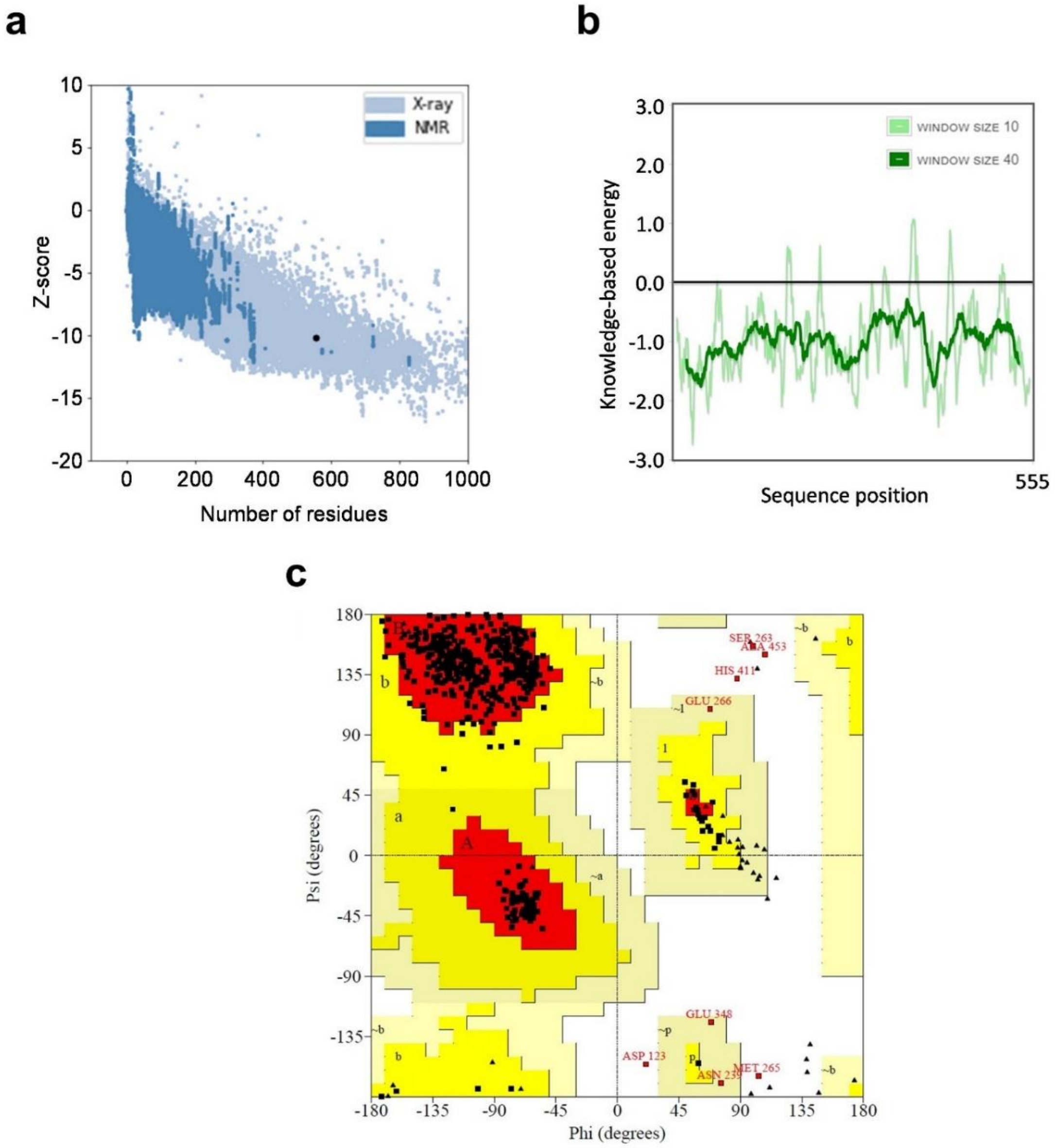

Suplementary Fig. 4: Assessment of the overall and local quality of the MFHR13 structure model. (a) Overall model quality of MFHR13 obtained by ProsaWEB. Z-score: -10.07. (b) Local model quality. Diagram of energy as a function of residues sequence position. Average energy over 40 or 10 amino acid fragments was calculated by ProsaWEB. (c) Ramachandran plot analysis by PROCHECK web-based tool of the model MFHR13. The most favored regions are colored red (labeled A, B, L), allowed regions are colored dark yellow (labeled a, b, l, p), and generously allowed regions are colored in shades of light yellow (labeled~a,~b,~l,~p), while amino acids in disallowed regions are indicated as red squares. The analysis revealed that 97.6, 1.3 and 1.1% of the residues are located in favored, allowed and outlier regions, respectively.

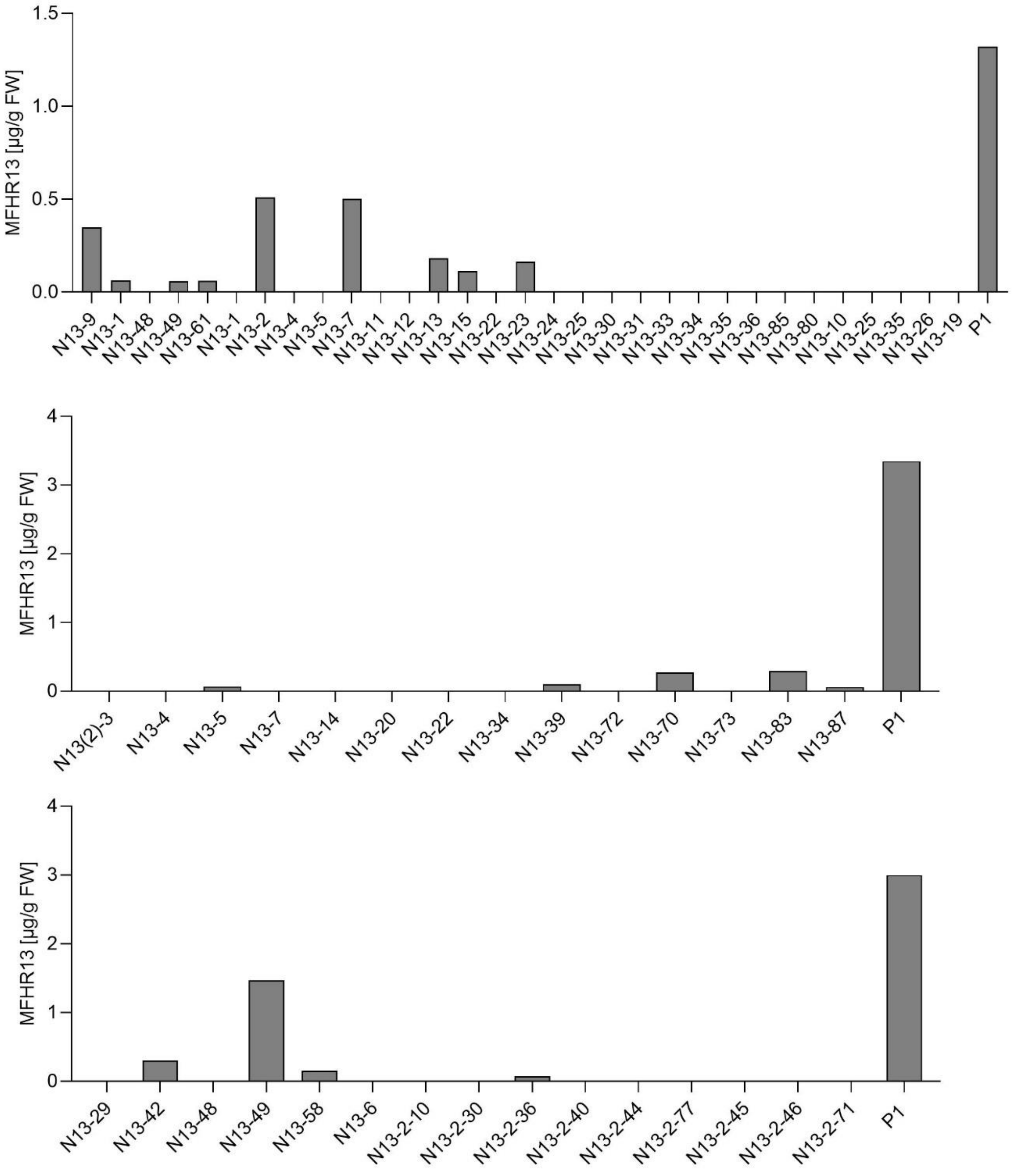

Supplementary Fig. 5: MFHR13 productivity. All plants surviving the selection were screened for productivity via ELISA in suspension cultures in agitated flasks. P1 is the MFHR1^{V62} moss producer line, which was included as an internal positive control.

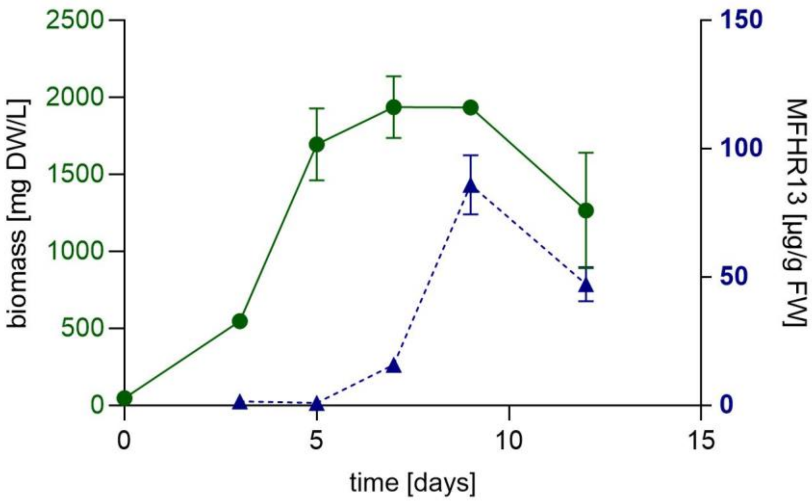

Supplementary Fig. 6: Kinetics of biomass accumulation and MFHR13 specific productivity of moss cell line N13-49 in a 5 L stirred bioreactor.

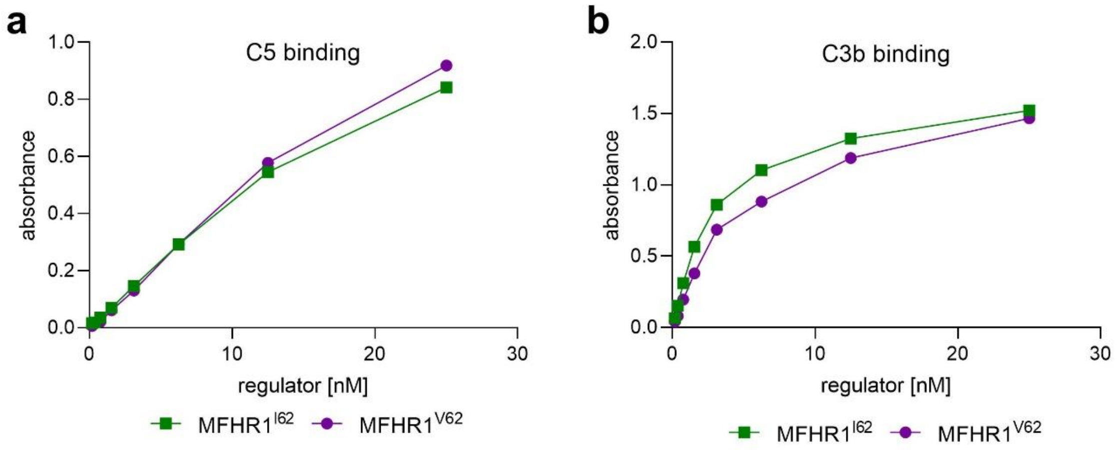

Supplementary Fig. 7: Evaluation of C5 and C3b binding to MFHR1^{I62} and MFHR1^{V62}. One representative experiment for the results presented in (a) Figure 2a and (b) 2b is shown with absolute magnitudes for the absorbance.

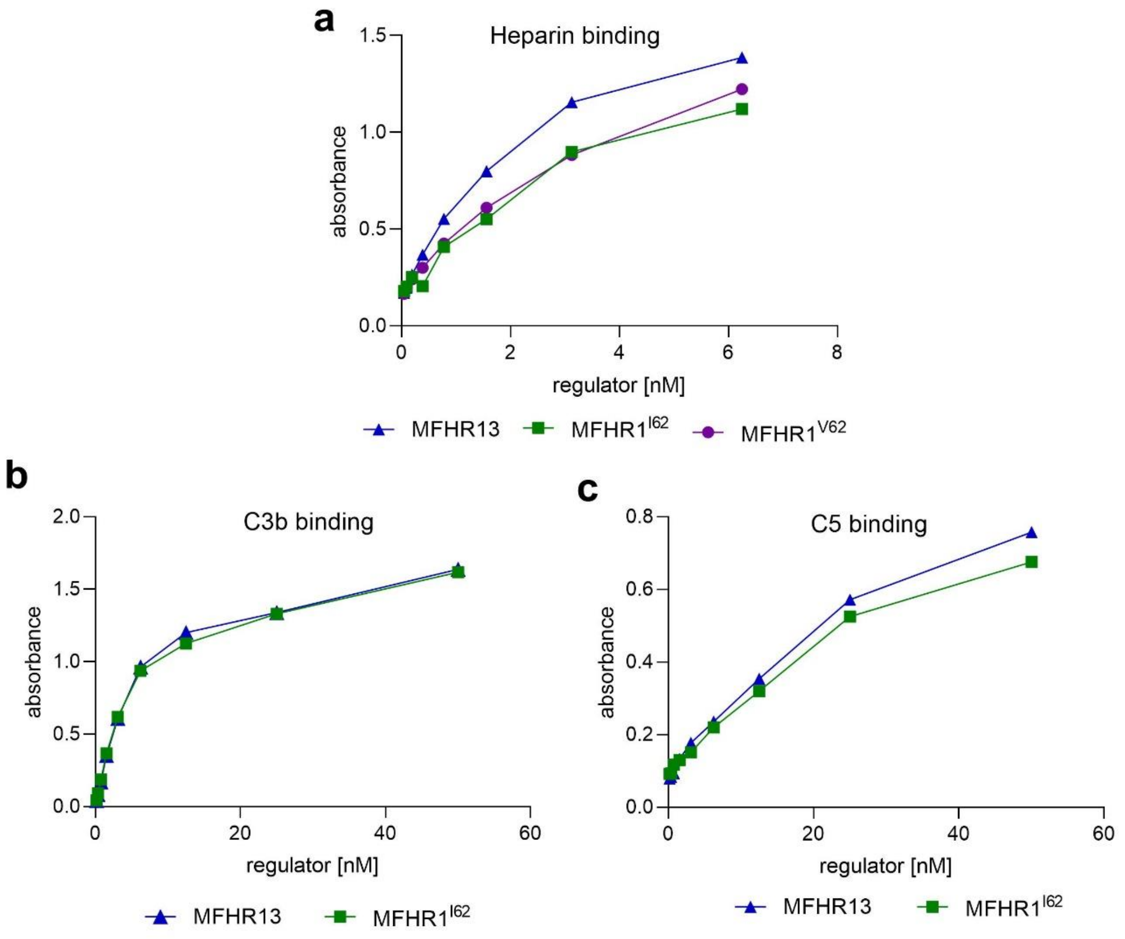

Supplementary Fig. 8: MFHR13 binds to heparin, C3b and C5. Binding was analyzed by ELISA and data from one representative experiment presented in (a) Figure 5a (b) Figure 6a (c) Figure 7a (c) is shown with absolute magnitudes for the absorbance.

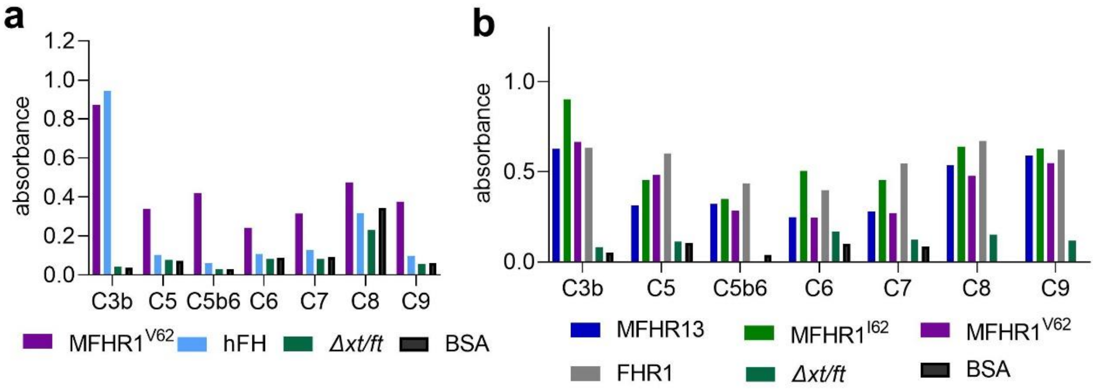

Supplementary Fig. 9: MFHR13 binds to the proteins of the terminal complement pathway. Binding was analyzed by ELISA and complement components were immobilized. Data from one representative experiment presented in Figure 7b and 7c are shown with absolute magnitudes for the absorbance. (a) MFHR1 binds to the TCC proteins but hFH does not. (b) MFHR13, MFHR1 and FHR1 bind to the TCC proteins.

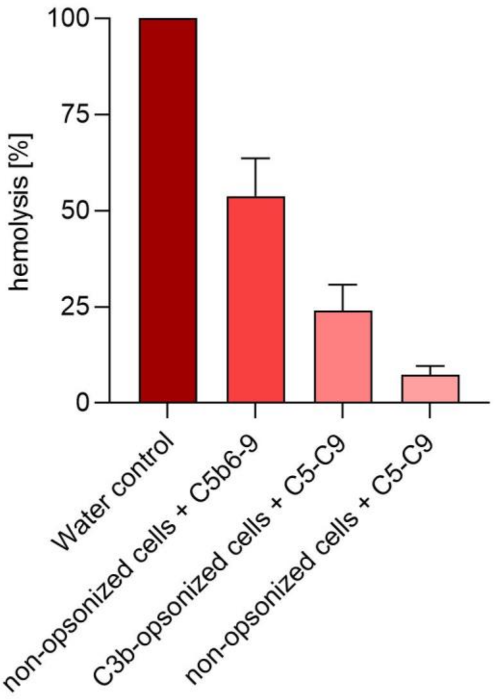

Supplementary Fig. 10: Comparison of hemolysis achieved by convertase-dependent and convertase-independent activation of C5. The extent of hemolysis achieved after activating C5 in the absence of convertases by using C3b-opsonized sheep erythrocytes exposed to C5-C9 mixture (equivalent to 20% normal human serum) is weaker than the C5b6-mediated hemolysis in non-opsonized erythrocytes. Additionally, a negative control of non-opsonized cells exposed to C5-C9 is included. Cells in water were set to 100%. Data represent mean values ± SD from three independent experiments.

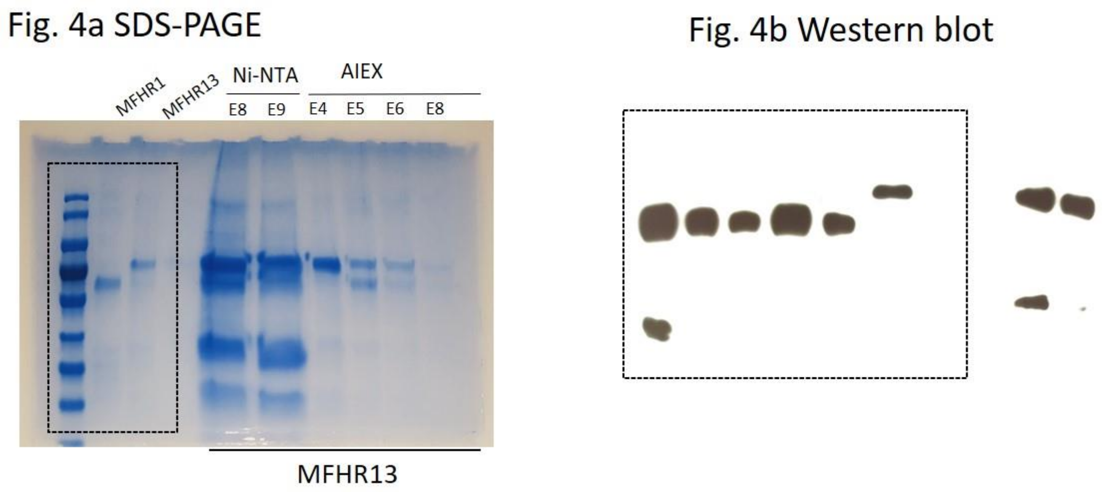

Supplementary Fig. 11: Uncropped scans shown in Fig. 4a, b.

Supplementary Table 1: Primers used to create the expression constructs pAct5-MFHR1^{I62} and pAct5-MFHR13, by directed mutagenesis and Gibson assembly respectively. Mismatch for codon exchange is shown in red and the overhangs (overlapping regions for Gibson assembly) in lowercase.

**MFHR1_I62_fwd**	TCTTGGAAATATAATAATGGTATGCAG
**MFHR1_I62_rev**	GATCTATATCCAGGGCGG
**SCR13_overlapSCR4_fwd**	gatggcgtccgttgccttcatgtGTGGCAATAGATAAACTTAAGAAG
**SCR13_overlapSCR19_rev**	tgtcaataggtggaggggcccacaTAATTGTATTTGTGCCATTG
**SCR4_overlapSCR13_rev**	cttcttaagtttatctattgccacACATGAAGGCAACGGACGC
**SCR19_overlapSCR13_fwd**	ctgctcaatggcacaaatacaattaTGTGGGCCCCTCCACC

Supplementary Table 2: Estimated IC_{50} values for C3b and C5 binding of MFHR1^{I62} and MFHR1^{V62} by fitting the data shown in Figure 2a,b with 4PL nonlinear regression model. CI: Confidence Interval, R^2: Goodness of Fit. Comparison of fits was carried out using the extra sum-of-squares F test C3b binding: P= 0.0437, F(DFn, Dfd)= 4,338 (1,40) C5 binding: P= 0.9328, F(DFn, Dfd)= 0.007207 (1,40). DF= degrees of freedom

	**MFHR1^{I62}**	**MFHR1^{V62}**
**C3b binding**		
IC_{50} (nM)	4.70	6.32
95% CI	3.82 - 5.80	5.88 - 6.80
R^2	0.99	0.99
**C5 binding**		
IC_{50} (nM)	26.83	26.83
95% CI	18.15 - 56.97	18.15 - 56.97
R^2	0.99	0.99

Supplementary Table 3: Estimated IC_{50} values for heparin binding of MFHR13 and MFHR1 variants fitting the data shown in Figure 5a with 4PL nonlinear regression model. CI: Confidence Interval, R^2: Goodness of Fit. Comparison of fits was carried out using the extra sum-of-squares F test. $P < 0.0001$, F(DFn, Dfd)= 29.76 (2, 94). MFHR13 vs MFHR1^{I62} $P < 0.0001$, F(DFn, Dfd) = 23.42 (1, 66). MFHR13 vs MFHR1^{V62} $P < 0.0001$, F(DFn, Dfd) = 42.53 (1, 67). DF= degrees of freedom,

**Heparin binding**	**MFHR13**	**MFHR1^{I62}**	**MFHR1^{V62}**
IC_{50} (nM)	0.24	0.50	0.76
95% CI	0.20 - 0.28	0.4 - 0.65	0.58 - 1.17
R^2	0.98	0.98	0.98

Supplementary Table 4: Estimated IC_{50} values for C3b and C5 binding of MFHR13 and MFHR1^{I62} by fitting the data shown in Figure 6a and 7a with 4PL nonlinear regression model. CI: Confidence Interval, R^2: Goodness of Fit. Comparison of fits was carried out using the extra sum-of-squares F test. C3b binding P= 0.8426 F(DFn, Dfd)= 0.0397 (1, 71). C5 binding P= 0.2565 F(DFn, Dfd)= 1.309 (1, 69). DF= degrees of freedom

**C3b binding**	**MFHR13**	**MFHR1^{I62}**
IC_{50} (nM)	16.05	16.05
95% CI	8.79 - 635.1	8.79 - 635.1
R^2	0.93	0.91
**C5 binding**		
IC_{50} (nM)	52.5	52.5
95% CI	Not calculated	Not calculated
R^2	0.86	0.96

Supplementary Table 5. IC_{50} calculated by 4PL nonlinear regression model for decay acceleration activity (DAA), fitting the data shown in Figure 6d. CI: Confidence Interval, R^2: Goodness of Fit. Comparison of fits was carried out using the extra sum-of-squares F test. MFHR13 vs MFHR^{I62} P<0.0001 DFn, DFd = 196.6 (1, 28). MFHR13 vs MFHR^{V62} P<0.0001 DFn, DFd = 131.5 (1, 31). MFHR13 vs hFH P=0.2022 F (DFn, DFd) 1.7 (1, 30). DF= degrees of freedom

	**MFHR13**	**MFHR1^{I62}**	**MFHR1^{V62}**	**hFH**
IC_{50} (nM)	2.70	7.8	9.45	3.84
95% CI	2.2 - 3.3	6.2 - 9.99	7.7 - 11.7	2.95 - 5.07
R^2	0.99	0.96	0.95	0.96
