# Supplementary material for: A synthetic protein as efficient multitarget regulator against complement over-activation
Source: Commun Biol. 2022 Feb 22;5:152. doi: 10.1038/s42003-022-03094-5 (PMC8863895; doi:10.1038/s42003-022-03094-5)
Supplement: Supplementary file 3 — Description of Additional Supplementary Files [file 42003_2022_3094_MOESM3_ESM.pdf]

## Description of Additional Supplementary Files

**File name:** Supplementary Data 1

**Description:** All source data underlying the graphs presented in the figures 2, 4, 5, 6, 7, and 8.
