# Supplementary material for: A synthetic protein as efficient multitarget regulator against complement over-activation
Source: Commun Biol. 2022 Feb 22;5:152. doi: 10.1038/s42003-022-03094-5 (PMC8863895; doi:10.1038/s42003-022-03094-5)
Supplement: Supplementary file 5 — Reporting Summary [file 42003_2022_3094_MOESM5_ESM.pdf]

nature research

Corresponding author(s): Eva L. Decker

Last updated by author(s): 2022.01.21

Reporting Summary

Nature Research wishes to improve the reproducibility of the work that we publish. This form provides structure for consistency and transparency in reporting. For further information on Nature Research policies, see our Editorial Policies and the Editorial Policy Checklist.

Statistics

For all statistical analyses, confirm that the following items are present in the figure legend, table legend, main text, or Methods section.

n/a	Confirmed	
☐	☒	The exact sample size (*n*) for each experimental group/condition, given as a discrete number and unit of measurement
☒	☐	A statement on whether measurements were taken from distinct samples or whether the same sample was measured repeatedly
☐	☒	The statistical test(s) used AND whether they are one- or two-sided<br>*Only common tests should be described solely by name; describe more complex techniques in the Methods section.*
☒	☐	A description of all covariates tested
☐	☒	A description of any assumptions or corrections, such as tests of normality and adjustment for multiple comparisons
☐	☒	A full description of the statistical parameters including central tendency (e.g. means) or other basic estimates (e.g. regression coefficient) AND variation (e.g. standard deviation) or associated estimates of uncertainty (e.g. confidence intervals)
☐	☒	For null hypothesis testing, the test statistic (e.g. *F*, *t*, *r*) with confidence intervals, effect sizes, degrees of freedom and *P* value noted<br>*Give P values as exact values whenever suitable.*
☒	☐	For Bayesian analysis, information on the choice of priors and Markov chain Monte Carlo settings
☒	☐	For hierarchical and complex designs, identification of the appropriate level for tests and full reporting of outcomes
☒	☐	Estimates of effect sizes (e.g. Cohen's *d*, Pearson's *r*), indicating how they were calculated

Our web collection on statistics for biologists contains articles on many of the points above.

Software and code

Policy information about availability of computer code

Data collection	no software was used for data collection
Data analysis	GraphPad Prism software version 8.0 for Windows (GraphPad software, San Diego, California, USA) for statistical analysis<br>Modeller 9.19 (Fiser & Šali, 2003) to predict the three-dimensional structure of the protein.<br>PyMOL Molecular Graphics System, Version 2.0, Schrödinger, LLC (PyMOL 2.0) for protein structure visualization<br>Ramchandran Plot SAVeS Server (PROCHECK) (https://services.mbi.ucla.edu/SAVES/), ProsaWEB (https://prosa.services.came.sbg.ac.at/prosa.php) to evaluate the quality of the model.<br>For glycosylation analysis the following softwares or database were used:<br>Mascot Distiller V2.7.10, Mascot server V2.7 (Matrix Science), Scaffold5 software (Proteome Software, Inc.) and MaxQuant (V1.6.0.16)

For manuscripts utilizing custom algorithms or software that are central to the research but not yet described in published literature, software must be made available to editors and reviewers. We strongly encourage code deposition in a community repository (e.g. GitHub). See the Nature Research guidelines for submitting code & software for further information.

Data

Policy information about availability of data

All manuscripts must include a data availability statement. This statement should provide the following information, where applicable:

- Accession codes, unique identifiers, or web links for publicly available datasets
- A list of figures that have associated raw data
- A description of any restrictions on data availability

All data generated in this study is included in this paper and the supplementary information (uncropped gel and blot images are shown in Supplementary Fig. 11). Additionally, a source data file is provided as Supplementary Data 1. Any remaining information can be obtained from the corresponding author upon reasonable

request. The mass spectrometry proteomics data have been deposited to the ProteomeXchange Consortium via the PRIDE partner repository with the dataset identifier PXD025471 and 10.6019/PXD025471

Field-specific reporting

Please select the one below that is the best fit for your research. If you are not sure, read the appropriate sections before making your selection.

☒ Life sciences ☐ Behavioural & social sciences ☐ Ecological, evolutionary & environmental sciences

For a reference copy of the document with all sections, see nature.com/documents/nr-reporting-summary-flat.pdf

Life sciences study design

All studies must disclose on these points even when the disclosure is negative.

Sample size	Sample size was not calculated through a statistical method.
Data exclusions	Experiments where non linear regression was applied, outliers were detected and removed by Prism using Robust regression and Outlier removal, however all data are presented in the figures.
Replication	Every experiment was reproduced independently. The number of repetitions, mean and standard deviation is presented in every figure.
Randomization	not applicable
Blinding	Blinding was not applicable because allocating data into groups was not included in this study

Reporting for specific materials, systems and methods

We require information from authors about some types of materials, experimental systems and methods used in many studies. Here, indicate whether each material, system or method listed is relevant to your study. If you are not sure if a list item applies to your research, read the appropriate section before selecting a response.

Materials & experimental systems

n/a	Involved in the study	
☐	☒	Antibodies
☐	☒	Eukaryotic cell lines
☒	☐	Palaeontology and archaeology
☒	☐	Animals and other organisms
☒	☐	Human research participants
☒	☐	Clinical data
☒	☐	Dual use research of concern

Methods

n/a	Involved in the study	
☒	☐	ChIP-seq
☒	☐	Flow cytometry
☒	☐	MRI-based neuroimaging

Antibodies

Antibodies used

- Complement Factor H Monoclonal Antibody (C18/3) (GAU 018-03-02, Thermo Fisher Scientific)
- polyclonal anti-FH1-4 antibody (Kühn et al., 1995)
- anti-FH polyclonal antibody (Oppermann et al., 2006)
- anti-His-tag antibody (Monoclonal Mouse IgG1) (MAB050, R&D Systems) and (ab18184, abcam)
- C9 neoepitope-specific antibodies (aE11, SantaCruz Biotechnologie)
- anti-factor B polyclonal antibody (341272, Merck)
- HRP-conjugated anti-mouse IgG sheep (NA931, Cytiva)
- HRP-conjugated anti-rabbit IgG from donkey (NA934; Cytiva)
- HRP-conjugated anti-goat from rabbit (P0448, Dako)

Validation

Polyclonal anti FH (SCR1-4) and polyclonal anti- FH were produced by Prof. Zipfel (Jena) and validated previously (Kühn et al., 1995 and Oppermann et al. (2006) respectively.

these antibodies were validated by the manufacturer
"GAU 018-03-02 has been successfully used in ELISA and Western blot procedures. GAU 018-03-02 can be used as a capture antibody in sandwich ELISA with GAU 020-03-02 as detection antibody. This antibody pair is specific for the ~150 kDa factor H protein". (https://www.thermofisher.com/antibody/product/Complement-Factor-H-Antibody-clone-C18-3-Monoclonal/GAU%2018-03-02)

anti-His-tag antibody (Monoclonal Mouse IgG1) (MAB050, R&D Systems): "Detects proteins containing accessible consecutive histidine regions. The antibody detects His tags localized at the amino- or carboxyl-terminus." (https://www.rndsystems.com/products/his-tag-antibody-ad1110_mab050)

C9 neoepitope-specific antibodies (aE11, SantaCruz Biotechnologie): mouse monoclonal (aE11) reactivity: human. application: ELISA, immunohistochemistry, immunocytochemistry

we evaluated different antibodies and standarized the dilutions for different binding ELISAs

Eukaryotic cell lines

Policy information about cell lines

Cell line source(s)	cell lines corresponding to Physcomitrella (Physcomitrium patens) were obtained from the International Moss Stock Center (https://www.moss-stock-center.org/en/)<br>- Δxt/ ft moss line: (IMSC no.: 40828)<br>- moss line P1 (IMSC no.: 40838)<br>The lines generated in this study were deposited in the IMSC:<br>- N-179 (IMSC no.: 40839)<br>- N13-49 (IMSC no.: 40840)
Authentication	none of the cell lines were authenticated
Mycoplasma contamination	we used moss cell lines, therefore they were not tested for mycoplasma contamination
Commonly misidentified lines (See ICLAC register)	we did not use commonly misdentified lines
